# Synthesis of quinoline-3-carboxylates by a Rh(II)-catalyzed cyclopropanation-ring expansion reaction of indoles with halodiazoacetates

**DOI:** 10.3762/bjoc.11.210

**Published:** 2015-10-20

**Authors:** Magnus Mortén, Martin Hennum, Tore Bonge-Hansen

**Affiliations:** 1 Department of Chemistry, University of Oslo, P.O. Box 1033 Blindern, NO-0315 Oslo, Norway

**Keywords:** catalysis, cyclopropanation, indole, quinoline, Rh(II), ring expansion

## Abstract

In this letter, we report a novel synthesis of ethyl quinoline-3-carboxylates from reactions between a series of indoles and halodiazoacetates. The formation of the quinoline structure is probably the result of a cyclopropanation at the 2- and 3-positions of the indole followed by ring-opening of the cyclopropane and elimination of H–X.

## Introduction

The indole moiety is found in a large number of bioactive natural products [1–2] and pharmaceutical compounds [3–4] and there has been a large synthetic effort going into the search for mild, efficient and selective procedures for the derivatization of indoles. One of the most efficient approaches is the transition metal-catalyzed C–H functionalization by diazo compounds [5–8]. The reactions of indoles with electrophilic metal-bound carbenes, or carbenoids, generated from diazo compounds, takes place under mild reaction conditions. The reaction has been studied for the three principle classes of carbenoids: acceptor-acceptor [9–11], mono-acceptor [12] and donor-acceptor [13–16], and all the carbenoids react preferentially at the electron rich C2–C3 double bond. The catalysts used for the generation of the carbenoids are typically salts of Cu [9,11,13], Rh [10,12,16], Fe [14] and Ru [15]. The substitution pattern of the indole substrate can have a significant effect on chemo- and regioselectivity. Electron-rich indoles typically give selective alkylation at the C3 position in the absence of a preexisting C3-substituent. In the presence of a C3-substituent, the alkylation is usually directed to the C2-position [5–8]. Electron-withdrawing groups (acyl, carbamoyl) at the indole nitrogen typically lead to cyclopropanation products that can be purified and isolated. Several reaction pathways have been proposed to account for the observed reactivity, but the mechanism for many of the reactions between indoles and metal-bound carbenes are unclear [5–8].

**Scheme 1:**
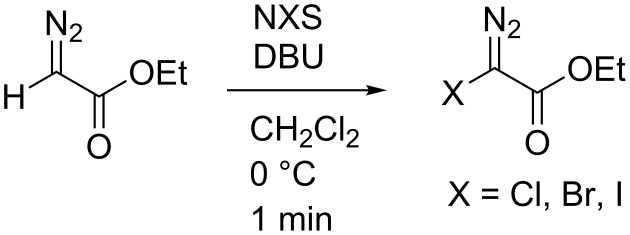
Synthesis of halo diazoacetates [17].

We have for some time worked with halodiazoacetates and studied their reactivity in cyclopropanation reactions [17], C–H insertion and Si–H insertion reactions [18]. Halodiazoacetates can be made quantiatively and rapidly (<1min reaction time) from ethyl diazoacetate (EDA), DBU and an *N*-halo succinimide (NXS) of choice according to Scheme 1.

In this letter we report our results from the reactions between a series of indoles with halodiazoacetates.

## Results and Discussion

**Scheme 2:**
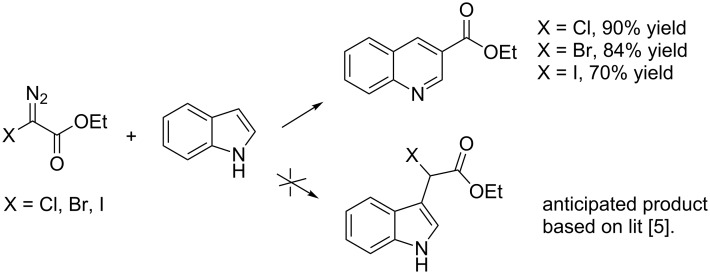
The reaction between halodiazoacetates and indole.

We initiated our study by investigating the reaction between ethyl bromodiazoacetate (Br-EDA) and indole (Scheme 2). We studied the reaction both thermally (room temperature, no catalyst) and in the presence of catalytic amounts of Rh_2_(esp)_2_ [19].

Indole is electron rich and will typically favor alkylation of the carbenoid at the 3-position [5–8]. Hence, the anticipated initial product from the reaction would be the bottom structure in Scheme 2. However, the only isolated product from both reactions was ethyl quinoline-3-carboxylate (top structure in Scheme 2). Even though we obtained the ethyl quinoline-3-carboxylate from the thermal (non-catalytic) reaction, ^1^H NMR analysis of the crude reaction mixtures showed greater conversion of indole and less byproducts for the catalyzed reaction compared to the thermal reaction. We optimized the catalytic reaction conditions and found that dropwise addition of an ice-cooled solution of Br-EDA (~1.4 equiv) to a room temperature solution of Rh_2_(esp)_2_ (1 mol %), Cs_2_CO_3_ (2.0 equiv) and indole (1.0 equiv) worked nicely. We used the optimized reaction conditions to study the reaction with three halodiazoacetates (X = Cl, Br, I). Ethyl quinoline 3-carboxylate was the isolated product from all three reactions and the yields were 90% (X = Cl), 84% (X = Br) and 70% (X = I).

The synthetic utility of the heterocyclic cyclopropanation-ring expansion reaction has been limited due to very low yields, strong basic reaction conditions, high temperature and formation of large amounts of polymeric byproducts [20–21]. In comparison, the Rh(II)-catalyzed reaction between indole and ethyl halodiazoacetates took place under mild reaction conditions and gave ethyl quinoline-3-carboxylate in good to high yields. Based on our promising results from the initial experiments we decided to investigate the scope and limitations of the cyclopropanation-ring expension reaction by treating a series of indoles with Br-EDA in the presence of catalytic amounts of Rh_2_(esp)_2_ and Cs_2_CO_3_ to neutralize HBr. The results are summarized in Table 1. The reaction worked well for a range of indoles. Both an electron donating group (OMe, Table 1, entry 3), an electron withdrawing group (NO_2_, Table 1, entry 4) as well as an halogen (Br, Table 1, entry 2) in indole’s 5-position were tolerated. The yield was excellent for the electron rich 5-MeO-indole (98%) and dropped off slightly for the 5-bromoindole (83%), while the 5-nitro derivative gave 69% yield. The position of the halogen substituent on the indole substrate had a significant effect on the efficiency of the reaction. The 4-bromoindole substrate gave a slightly lower yield (71%) compared to 5-bromoindole while the 6-bromoindole gave an excellent yield (94%). The reaction was sluggish and did not go to completion with 7-chloroindole. Even after increasing to two equivalents of Br-EDA, there was still unreacted 7-chloroindole left in the crude reaction mixture and the yield was only 40% (Table 1, entry 7). We also performed the reaction with 7-nitroindole (not shown), but the conversion was only ~10%, confirming that indoles with a substituent in the 7-position are poor substrates for this reaction. Methyl 3-indolylacetate (Table 1, entry 8) was a very intereresting substrate as a probe for the scope and limitations of the cyclopropanation-ring expansion since the substitution pattern of an indole substrate, particularly in the 2- and 3-position, can have a significant effect on the reactivity and selectivity. The reaction gave a good yield (70%) of the 4-substituted quinoline carboxylate, demonstrating that a substituent in indole’s 3-position was tolerated well. The second limitation we found among the screened substrates was the lack of reactivity for indole substituted in the 2-position. The 2-methylindole starting material did not participate in the reaction and was left unchanged after all Br-EDA was fully consumed (Table 1, entry 9). We repeated the reaction with 2-methylindole and Br-EDA in the absence of the Rh(II) catalyst and found that the thermal reaction gave the corresponding quinoline structure, but the conversion of 2-methylindole was still low and the yield was poor (~15%, measured by internal standard, ^1^H NMR).

**Table 1:** Substrate scope.

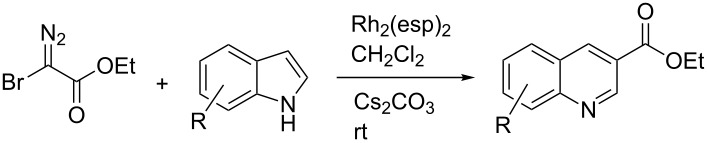			

Entry	Substrate	Product	Yield (%)^a^

1	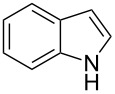	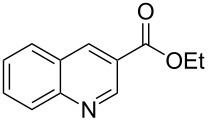	84
2	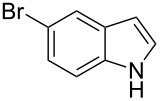	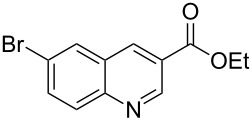	83
3	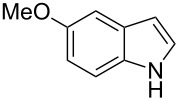	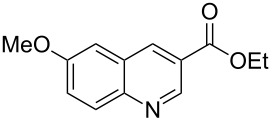	98
4	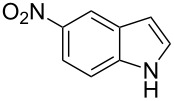	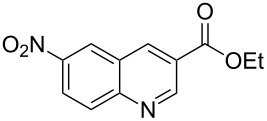	69
5	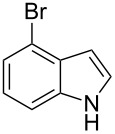	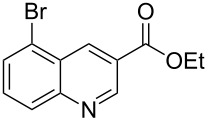	71
6	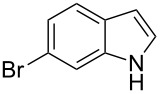	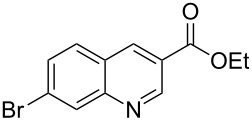	94
7	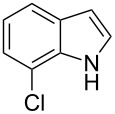	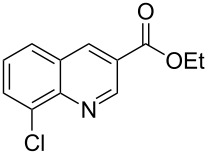	40^b^
8	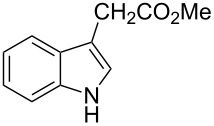	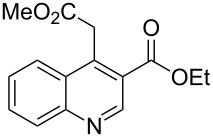	70
9	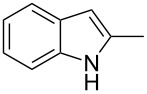	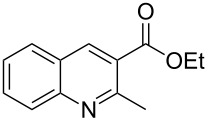	–

^a^Isolated yield. ^b^Measured by internal standard using ^1^H NMR.

The yields of ethyl quinoline 3-caboxylates we obtained by using our method (Table 1) compares favorably with three recently published methods where arylmethyl azides [22], arylallyl azides [23] and *o*-nitrobenzaldehydes [24] were used as starting materials.

**Scheme 3:**
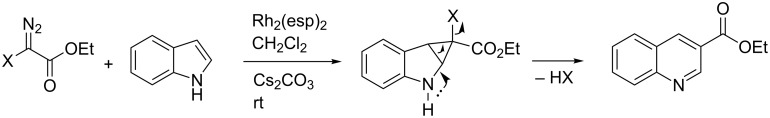
Proposed reaction pathway.

The mechanism of heterocyclic ring expansions has briefly been studied for thermal reactions between pyrrole and chloromethylcarbene, and 2,3-dimethylindole and dichloromethylcarbene [20–21]. It was suggested that the quinoline ring system is formed by ring expension of a labile indoline cyclopropane intermediate. In analogy to the postulated literature pathway, we propose that the reactions in our study start by cyclopropanation of the rhodium carbenoid to produce an indoline halocyclopropyl ester. The labile indoline intermediate then undergoes ring opening of the cyclopropane and elimination of H–X to form the quinoline structure (Scheme 3).

**Scheme 4:**
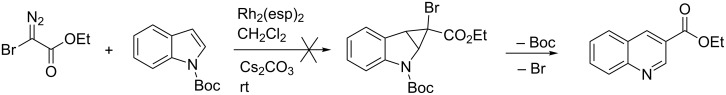
Attempted cyclopropanation of *N*-Boc indole.

We attempted to find support for the proposed reaction pathway by using *N*-Boc-indole as a substrate (Scheme 4). Reactions between *N*-Boc-indole and carbenoids typically give *N*-Boc-indoline cyclopropanation products that can be purified and isolated [5–8].

We exposed Br-EDA to Rh_2_(esp)_2_ in the presence of *N*-Boc-indole in order to obtain and isolate the analogous *N*-Boc-indolinebromocyclopropyl ester. If the cyclopropanation was successful, removal of the *N*-Boc protecting group would then give the labile indolebromocyclopropyl ester from the proposed reaction pathway in Scheme 3. Once the labile indolebromocyclopropyl ester is formed, a ring expansion and elimination of bromide would then give the quinoline structure. However, *N*-Boc-indole was a very poor substrate for the attempted cyclopropanation and no trace of the expected *N*-Boc-indolinebromocyclopropane was detected.

**Scheme 5:**
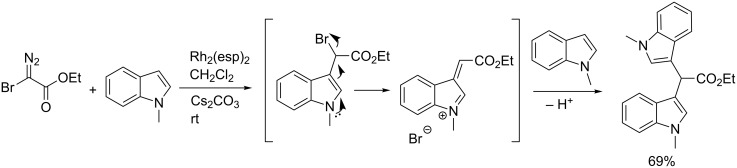
The reaction between ethyl bromo diazoacetate and *N*-Me-indole.

In all the substrates listed in Table 1, there is an N–H proton present for deprotonation and elimination of HBr. We wanted to investigate if the presence of the N–H proton was required for the cyclopropanation-ring expansion to take place. We selected *N*-methylindole as a substrate and used the optimized reaction conditions to study the reaction displayed in Scheme 5.

We found no trace of a cyclopropanation-ring expansion product in the crude reaction mixture, but isolated ethyl 2,2-bis(1-methyl-1*H*-indol-3-yl)acetate in 69% yield. A possible explanation for the formation of the bisindol acetate can be rationalized by assuming a C–H insertion of the Rh-carbenoid in the C3-position of *N*-methylindole followed by elimination of bromide. The conjugated iminium ion is a very good electrophile and can undergo an electrophilic aromatic substitution in the C3-position of *N*-methylindole to form the bisindole product.

## Conclusion

We have developed a mild and efficient method for the synthesis of ethyl quinoline-3-carboxylates from reactions between indoles and ethyl halodiazoacetates. The reaction propably follows a cyclopropanation-ring expansion pathway. Indoles with substituents in the 3,4,5 and 6 positions were good substrates for the reaction, but a subsituent in indole’s 7-position gave a poor reaction while a substituent in the 2-postion was detrimental. The presence of an indole N–H seems to be necessary for the cyclopropanation-ring expansion to occur since an *N*-substituted indole followed a different reaction pathway. The large selection of commercially available indoles in combination with the scope of the reaction makes the cyclopropanation-ring expansion an attractive method for an efficient synthesis of quinolone-3-carboxylates.

## Experimental

Detailed experimental procedures and analytical data for the compounds are available in Supporting Information File 1.

**General procedure for the synthesis of ethyl quionline-3-carboxylates**: An ice-cooled solution of X-EDA (1.4 mmol) in CH_2_Cl_2_ was added dropwise to a stirring solution (room temperature) of indole (1.0 mmol), Cs_2_CO_3_ (1.3 mmol) and Rh_2_(esp)_2_ (0.01 mmol) in CH_2_Cl_2_ (10 mL). After the addition was complete, the solution was stirred for 30 min and the solvent removed in vacuo. The crude product was dissolved in EtOAc and washed with water and brine. The organic phase was dried (MgSO_4_), filtered and the solvent removed in vacuo. The pure ethyl quinoline-3-carboxylate was isolated after purification over silica gel eluting with CH_2_Cl_2_/EtOAc.

## Supporting Information

File 1 Experimental procedures and characterization of compounds.
