# Supplementary material for: Synthesis of quinoline-3-carboxylates by a Rh(II)-catalyzed cyclopropanation-ring expansion reaction of indoles with halodiazoacetates
Source: Beilstein J Org Chem. 2015 Oct 20;11:1944–9. doi: 10.3762/bjoc.11.210 (PMC4661008; doi:10.3762/bjoc.11.210)
Supplement: File 1 — Experimental procedures and characterization of compounds. [file Beilstein_J_Org_Chem-11-1944-s001.pdf]

Supporting Information

for

Synthesis of quinoline-3-carboxylates by a Rh(II)-catalyzed cyclopropanation-ring expansion reaction of indoles with halodiazoacetates

Magnus Mortén, Martin Hennum and Tore Bonge-Hansen*

Address: Department of Chemistry, University of Oslo, P.O. Box 1033 Blindern, NO-0315 Oslo, Norway.

Email: Tore Bonge-Hansen* - tore.hansen@kjemi.uio.no

*Corresponding author

Experimental procedures and characterization of compounds

General information

All chemical were used as received from Sigma-Aldrich. NBS and NCS were recrystallized and dried under high vacuum prior to use.

Dichloromethane was dried using an MBraun MB-SPS800 solvent purification system.

Flash chromatography was performed on an Isco Inc. CombiFlash Companion system with silica gel from Fluka (60Å, 35–70 µm, high purity, pH 7). Eluents ethyl acetate (EtOAc) and dichlorometane (DCM) of technical grade were used.

Thin layer chromatography (TLC) was peformed on silica gel coated aluminium sheets from Merck (TLC silica gel, 60 F254).

NMR spectra were recorded on a Bruker AVII400 at room temperature at 400 MHz for ^{1}H NMR and 100 MHz for ^{13}C NMR with $CDCl_3$ as solvent, calibrated to 7.24 ppm for ^{1}H NMR and 77.00 ppm for ^{13}C NMR. Chemical shifts (δ) are given in ppm relative to the solvent $CDCl_3$.

MS spectra (EI) were recorded on a VG Prospec sector instrument from Fissions Instruments at 70 eV. High resolution masses (EI) were measured on a Micromass Q-Tof-2 mass spectrometer.

The structures of ethyl quinoline-3-carboxylate, ethyl 6-nitroquinoline-3-carboxylate and ethyl 4-(2-methoxy-2-oxoethyl)quinoline-3-carboxylate were confirmed by X-ray diffraction crystallography measured on a Bruker APEXII CCD diffractometer at room temperature.

All new compounds are fully characterized by NMR as well as MS (EI) and HRMS (ESI).
Previously reported compounds are characterized by ^{1}H NMR and MS (EI) and compared to the literature data given as references.

When the reactions were carried out with the halodiazoacetate as the limiting reagent and the indole substrate in excess, we found it very difficult to separate the quinoline product from the indole starting material by silica gel column chromatography. When the indole substrate was used as the limiting reagent and the halodiazoacetate was added in a small excess (~1.4 equivalents), we observed full conversion of the indole substrate and the quinoline product was easily obtained in high purity after silica gel chromatography.

General procedure for the synthesis of halodiazoacetates (X-EDA)

EDA (1.0 mmol) was diluted with CH_2Cl_2 (10 mL) and the solution cooled to 0 °C. To this solution was added DBU (1,4 mmol) and stirring was continued at 0 °C for 5 min before the *N*-halosuccinimide (1.1 mmol, NBS, NCS or NIS) of choice was added. There was an immediate color change from yellow to red, and the conversion of EDA was complete in less than 5 min judged by TLC analysis. After stirring for 5 min. at 0 °C, the solution was quickly filtered through a pre-cooled (0 °C) plug (2-3 cm) of silica gel, eluting with cold CH_2Cl_2. This gave X-EDA (X = Cl, Br or I depending on the choice of *N*-halosuccinimide) as an orange/red solution in CH_2Cl_2 (typically ~1 mmol in 50 ml). If desired, the volume of CH_2Cl_2 could be reduced to 10 ml in vacuo at 0 °C without any significant decomposition of the halodiazoacetate.

General procedure for the synthesis ethyl quinoline-3-carboxylates

The cooled solution of X-EDA (1.4 mmol) in CH_2Cl_2 was transferred to an ice-cooled addition funnel and added dropwise to a stirring solution (room temperature) of indole (1.0 mmol), Cs_2CO_3 (1.3 mmol) and $Rh_2(esp)_2$ (0.01 mmol) in CH_2Cl_2 (10 mL). After the addition was complete, the solution was stirred for 30 min and the solvent removed in vacuo. The crude product was dissolved in EtOAc and washed with water and brine. The organic phase was dried ($MgSO_4$), filtered and the solvent removed in vacuo. The pure ethyl quinoline-3-carboxylate was isolated after purification over silica gel eluting with CH_2Cl_2/EtOAc.

The source of diazo compound is given in parenthesis for the reported yields of the ethyl quinoline carboxylates.

Ethyl quinoline-3-carboxylate

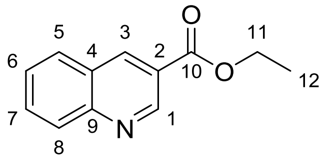

Yield: (Cl-EDA) = 90%, (Br-EDA) = 84% and (I-EDA) = 70%.

^{1}H-NMR (400 MHz; $CDCl_3$): δ 1.44 (t, *J* = 7.1 Hz, 3H, H12), 4.46 (q, *J* = 7.1 Hz, 2H, H11), 7.62-7.68 (m, 1H, H6), 7.79-7.83 (m, 1H, H7), 7.92 (d, *J* = 8.8 Hz, 1H, H5), 8.15 (d, *J* = 8.8 Hz, 1H, H8), 8.82 (d, *J* = 1.8 Hz, 1H, H3), 9.43 (d, *J* = 1.8 Hz, 1H, H1).

^{13}C-NMR (100 MHz; $CDCl_3$): δ 14.3 (C12), 61.5 (C11), 123.3 (C2), 126.9 (C6), 127.4 (C4), 129.1 (C5), 129.4 (C8), 131.8 (C7), 138.7 (C3), 149.7 (C1), 150.0 (C9), 165.3 (C10).

MS (EI) *m/z* (relative intensity): 201 (M^+, 74%), 156 (100), 173 (34), 128 (62). **HR-MS:** 201.0795; Calc. value for $C_{12}H_{11}NO_2$: 201.0790 (-2.4 ppm).

Mp: 62-65 °C. Lit: 69-70 °C.[1]

This compound has previously been reported in the literature. [1]

Ethyl 6-methoxyquinoline-3-carboxylate

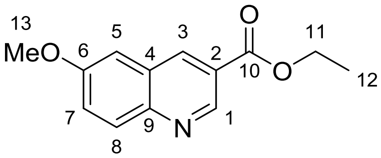

Deviation from the standard procedure: The solution of 5-MeO-indole, Cs_2CO_3 and $Rh_2(esp)_2$ in CH_2Cl_2 was kept at −78 °C during the addition of the Br-EDA solution and then warmed to rt.

Yield (Br-EDA) = 98%.

^{1}H-NMR (400 MHz; $CDCl_3$): δ 1.43 (t, *J* = 7.1 Hz, 3H, H12), 3.93 (s, 3H, H13), 4.45 (q, *J* = 7.1 Hz, 2H, H11), 7.14 (d, *J* = 2.8 Hz, 1H, H5), 7.45 (dd, *J* = 9.2, 2.8 Hz, 1H, H7), 8.04 (d, *J* = 9.2 Hz, 1H, H8), 8.72 (d, *J* = 2.1 Hz, 1H, H3), 9.28 (d, *J* = 2.1 Hz, 1H, H1).

^{13}C-NMR (100 MHz; $CDCl_3$): δ 14.3 (C12), 55.6 (C13), 61.4 (C11), 106.0 (C5), 123.5 (C2), 124.7 (C7), 128.0 (C4), 130.7 (C8), 137.3 (C3), 145.9 (C9), 147.6 (C1), 158.3 (C6), 165.5 (C10).

MS (EI) *m/z* (relative intensity): 231 (M^+, 100%), 203 (21), 186 (54), 158 (34). **HR-MS:** 231.0894; Calc. value for $C_{13}H_{13}NO_3$: 231.0895 (0.7 ppm).

Mp: 77-80 °C. Lit: 70-71 °C.[2]

This compound has previously been reported in the literature.[2]

Ethyl 6-nitroquinoline-3-carboxylate

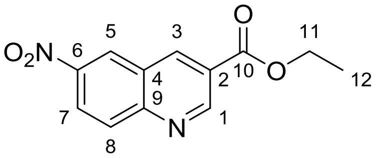

Yield (Br-EDA) = 69%

^{1}H-NMR (400 MHz; $CDCl_3$): δ 1.47 (t, *J* = 7.1 Hz, 3H, H12), 4.50 (q, *J* = 7.1 Hz, 2H, H11), 8.30 (d, *J* = 9.3 Hz, 1H, H8), 8.57 (dd, *J* = 9.3, 2.5 Hz, 1H, H7), 8.89 (d, *J* = 2.5 Hz, 1H, H5), 9.00 (d, *J* = 2.1 Hz, 1H, H3), 9.59 (d, *J* = 2.1 Hz, 1H, H1).

^{13}C-NMR (100 MHz; $CDCl_3$): δ 14.3 (C12), 62.1 (C11), 124.9 (C7), 125.1 (C2), 125.6 (C5), 125.9 (C4), 131.5 (C8), 140.1 (C3), 146.1 (C9), 151.6 (C6), 153.4 (C1), 164.4 (C10).

MS (EI) *m/z* (relative intensity): 246 (M^+, 76%), 218 (69), 201 (100), 173 (36).

HR-MS: 246.0638; Calc. value for $C_{12}H_{10}N_2O_4$: 246.0641 (1.1 ppm).

Mp: 185-188 °C. Lit: 186-187 °C.[2]

This compound has previously been reported in the literature.[2]

Ethyl 6-bromoquinoline-3-carboxylate

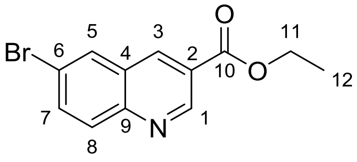

Yield (Br-EDA) = 83%

^{1}H-NMR (400 MHz; $CDCl_3$): δ 1.44 (t, *J* = 7.1 Hz, 3H, H12), 4.47 (q, *J* = 7.1 Hz, 2H, H11), 7.87 (dd, *J* = 9.0, 2.2 Hz, 1H, H7), 8.02 (d, *J* = 9.0 Hz, 1H, H8), 8.08 (d, *J* = 2.2 Hz, 1H, H5), 8.73 (d, *J* = 1.8 Hz, 1H, H3), 9.43 (d, *J* = 1.8 Hz, 1H, H1).

^{13}C-NMR (100 MHz; $CDCl_3$): δ 14.3 (C12), 61.7 (C11), 121.4 (C6), 124.1 (C2), 128.0 (C4), 130.9 (C5), 131.2 (C8), 135.1 (C7), 137.5 (C3), 148.4 (C9), 150.4 (C1), 165.0 (C10).

MS (EI) *m/z* (relative intensity): 279/281 ($[M/M+2]^+$, 100/98), 251/253 (36/35), 234/236 (88/87), 206/208(45/45).
HR-MS: 278.9905; Calc. value for $C_{12}H_{10}{}^{79}BrNO_2$: 278.9895 (-3.8 ppm).

Mp: 110-112 °C. Lit: 113-114.[1]

This compound has previously been reported in the literature.[1]

Ethyl 5-bromoquinoline-3-carboxylate

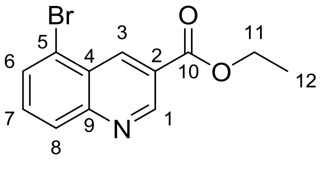

Yield (Br-EDA) = 71%

^{1}H-NMR (400 MHz; $CDCl_3$): δ 1.46 (t, *J* = 7.1 Hz, 3H, H12), 4.49 (q, *J* = 7.1 Hz, 2H, H11), 7.64-7.68 (m, 1H, H7), 7.87-7.89 (m, 1H, H6), 8.10-8.13 (m, 1H, H8), 9.16-9.18 (m, 1H, H3), 9.44 (d, *J* = 2.0 Hz, 1H, H1).

^{13}C-NMR (100 MHz; $CDCl_3$): δ14.4 (C12), 61.8 (C11), 123.2 (C5), 124.5 (C2), 126.6 (C4), 129.4 (C8), 131.2 (C6), 131.9 (C7), 138.1 (C3), 150.6 (C9), 150.7 (C1), 165.0 (C10).

MS (EI) *m/z* (relative intensity): 279/281 ($[M/M+2]^+$, 100/99), 251/253 (33/32), 234 (94), 206 (39).
HR-MS: 278.9889; Calc. value for $C_{12}H_{10}{}^{79}BrNO_2$: 278.9895 (2.2 ppm).

Mp: 84-86 °C.

This compound has not been previously reported in the literature with physical data.

Ethyl 7-bromoquinoline-3-carboxylate

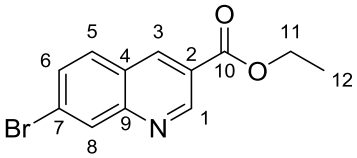

Yield (Br-EDA) = 94%

^{1}H-NMR (400 MHz; $CDCl_3$): δ 1.44 (t, *J* = 7.1 Hz, 3H, H12), 4.46 (q, *J* = 7.1 Hz, 2H, H11), 7.70 (dd, *J* = 8.7, 1.9 Hz, 1H, H6), 7.79 (d, *J* = 8.7 Hz, 1H, H5), 8.35 (s, 1H, H8), 8.80 (d, *J* = 2.1 Hz, 1H, H3), 9.43 (d, *J* = 2.1 Hz, 1H, H1).

^{13}C-NMR (100 MHz; $CDCl_3$): δ 14.3 (C12), 61.7 (C11), 123.7 (C2), 125.5 (C4), 126.4 (C7), 130.2 (C5), 131.2 (C6), 131.8 (C8), 138.6 (C3), 150.0 (C9), 150.9 (C1), 165.0 (C10).

MS (EI) *m/z* (relative intensity): 279/281 ($[M/M+2]^+$, 87/85), 251/253 (40/39), 234/236 (100/98), 206/208 (47/46).
HR-MS: 278.9889; Calc. value for $C_{12}H_{10}{}^{79}BrNO_2$: 278.9895 (2.1 ppm)

Mp: 94-96 °C. Lit: 98-99 °C.[1]

This compound has previously been reported in the literature.[1]

Ethyl 4-(2-methoxy-2-oxoethyl)quinoline-3-carboxylate

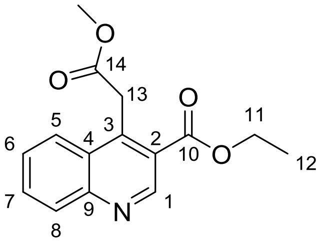

Yield (Br-EDA) = 70%

^{1}H-NMR (400 MHz; $CDCl_3$): δ 1.43 (t, *J* = 7.1 Hz, 3H, H12), 3.69 (s, 3H, H15), 4.44 (q, *J* = 7.1 Hz, 2H, H11), 4.62 (s, 2H, H13), 7.61-7.65 (m, 1H, H6), 7.77-7.81 (m, 1H, H7), 8.05- 8.11 (m, 1H, H5), 8.12-8.17 (m, 1H, H8), 9.37 (s, 1H, H1).

^{13}C-NMR (100 MHz; $CDCl_3$): δ 14.2 (C12), 34.1 (C13), 52.4 (C15), 61.7 (C11), 123.2 (C2), 124.5 (C5), 127.2 (C3), 127.7 (C6), 130.4 (C8), 131.1 (C7), 143.0 (C4), 149.3 (C9), 150.6 (C1), 166.2 (C10), 170.3 (C14).

MS (EI) *m/z* (relative intensity): 273 (M^+, 39%), 242 (18), 227 (100), 200 (39).
HR-MS: 273.1003; Calc. value for $C_{15}H_{15}NO_4$: 273.1001 (-0.8 ppm).

Mp: 82-84 °C.

This compound has not been previously reported in the literature.

Ethyl 2,2-bis(1-methyl-1*H*-indol-3-yl)acetate

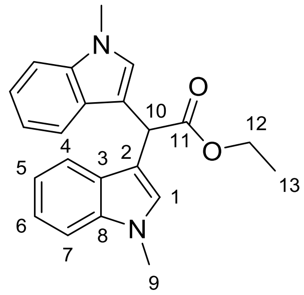

Yield (Br-EDA) = 69%

^{1}H-NMR (600 MHz; $CDCl_3$): δ 1.27 (t, *J* = 7.1 Hz, 3H, H13), 3.71 (s, 3H, H9), 4.22 (q, *J* = 7.1 Hz, 2H, H12), 5.50 (s, 1H, H10), 7.03 (s, 2H, H1), 7.08-7.11 (m, 2H, H5), 7.20-7.24 (m, 2H, H6), 7.28-7.30 (m, 2H, H7), 7.64-7.66 (m, 2H, H4).

^{13}C-NMR (150 MHz; $CDCl_3$): δ 14.3 (C13), 32.7 (C9), 40.4 (C10), 61.0 (C12), 109.2 (C7), 112.3 (C3), 119.0 (C5), 119.4 (C4), 121.6 (C6), 127.1 (C2), 127.9 (C1), 137.1 (C8), 173.5 (C11).

MS (EI) *m/z* (relative intensity): 346 (M^+, 13%), 273 (100).
HR-MS: 346.1678; Calc. value for $C_{22}H_{22}N_2O_2$: 346.1681 (0.9 ppm)

This compound has previously been reported in the literature.[3]

^{1}H NMR and ^{13}C NMR spectra

Ethyl quinoline-3-carboxylate. ^{1}H NMR ($CDCl_3$, 400 MHz) and ^{13}C NMR (100 MHz).

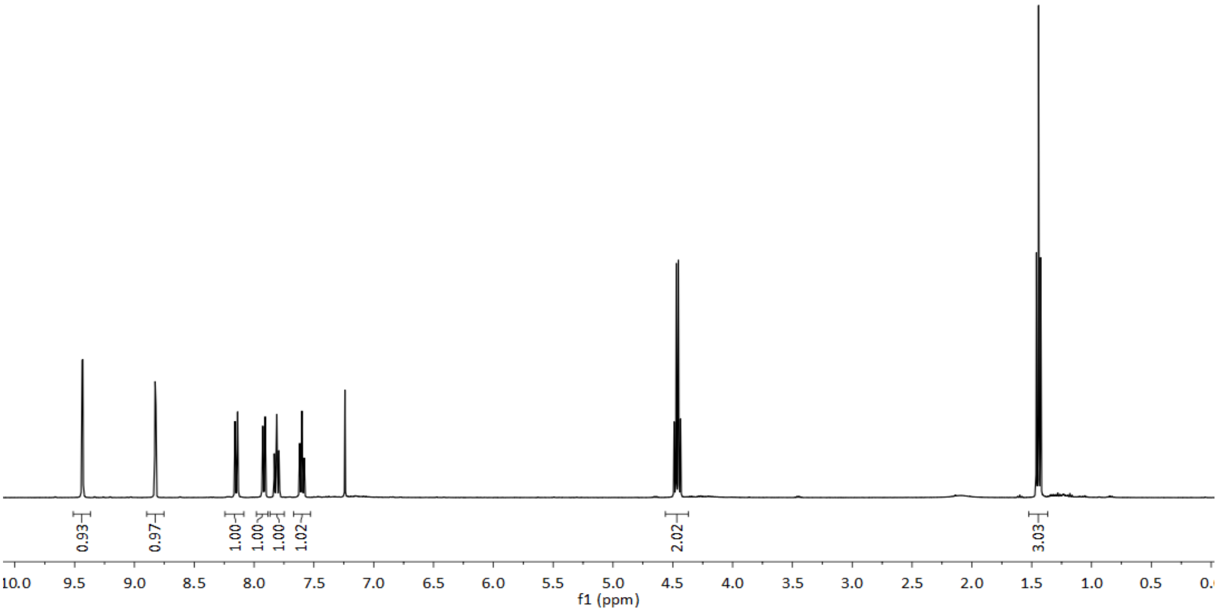

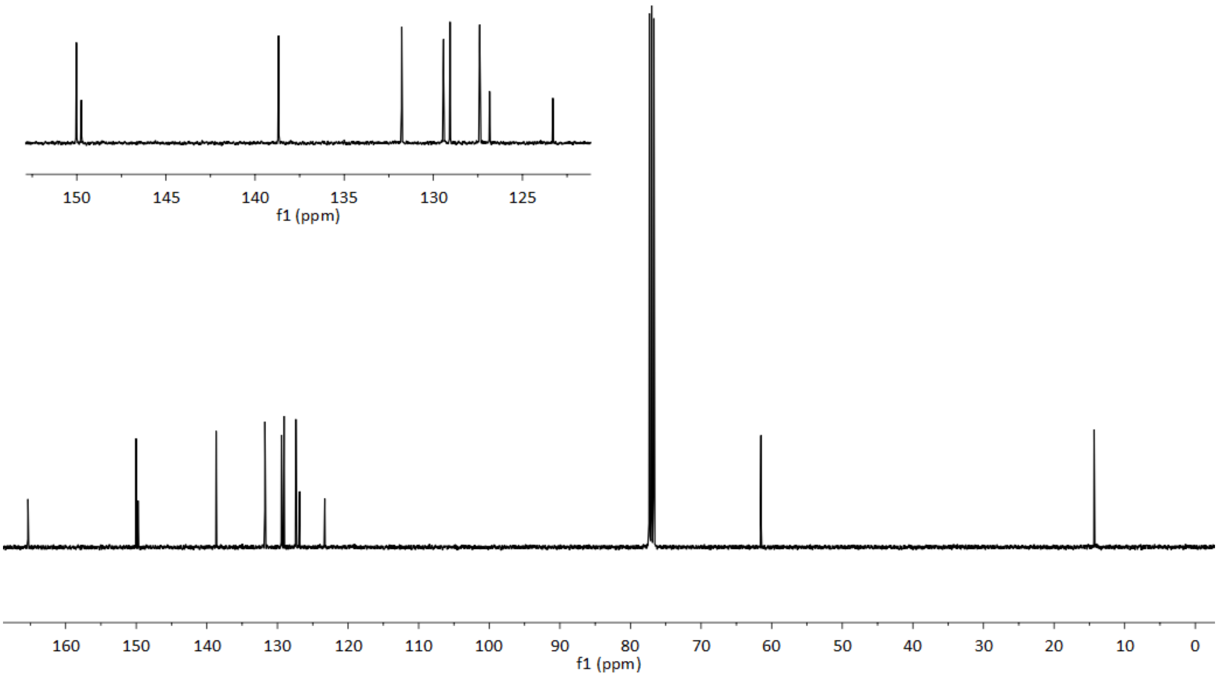

Ethyl 6-methoxyquinoline-3-carboxylate. ^{1}H NMR ($CDCl_3$, 400 MHz) and ^{13}C NMR (100 MHz).

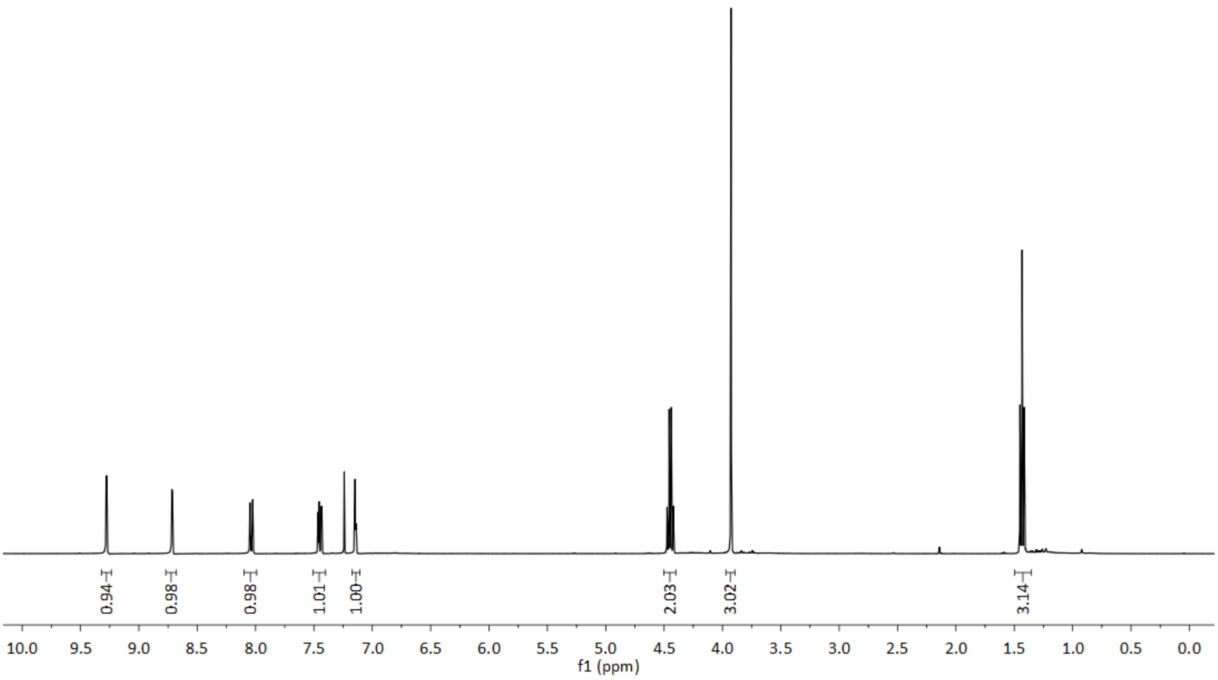

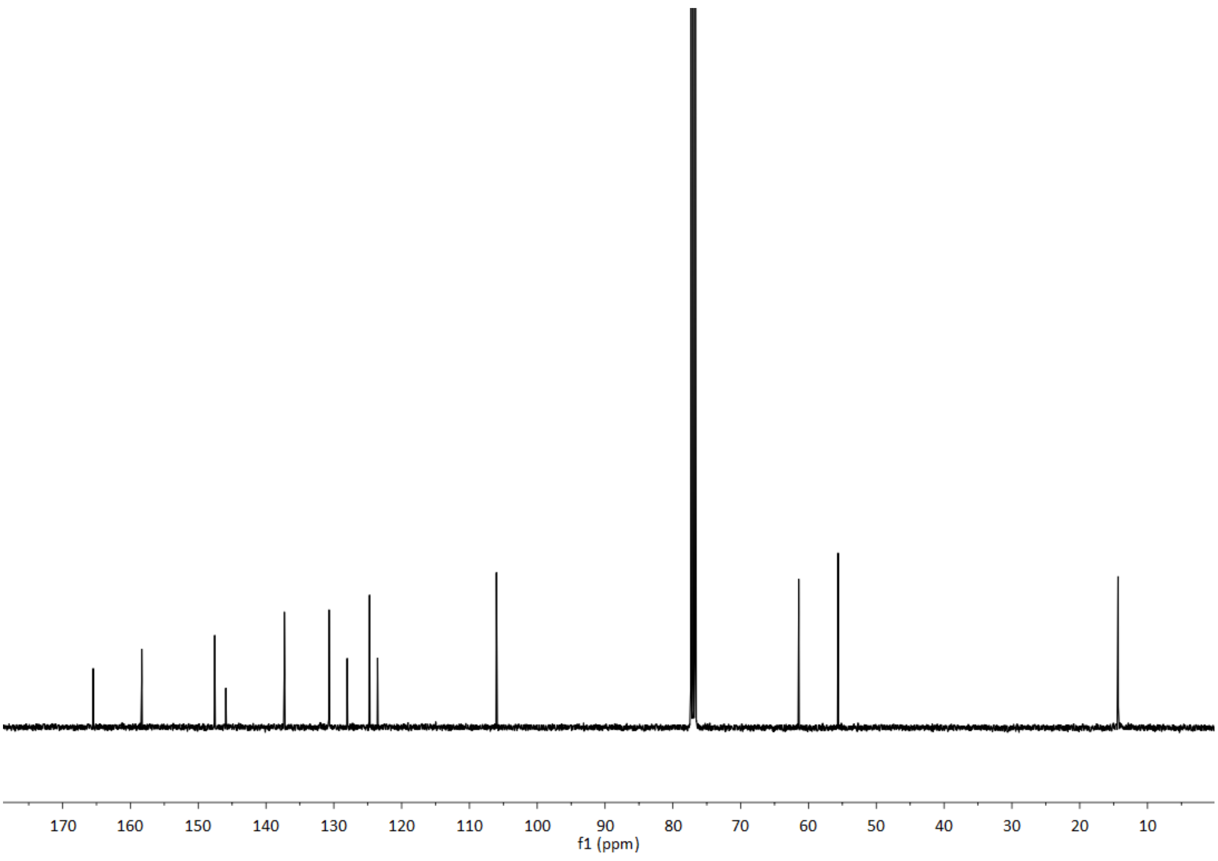

Ethyl 6-nitroquinoline-3-carboxylate. ^{1}H NMR ($CDCl_3$, 400 MHz) and ^{13}C NMR (100 MHz).

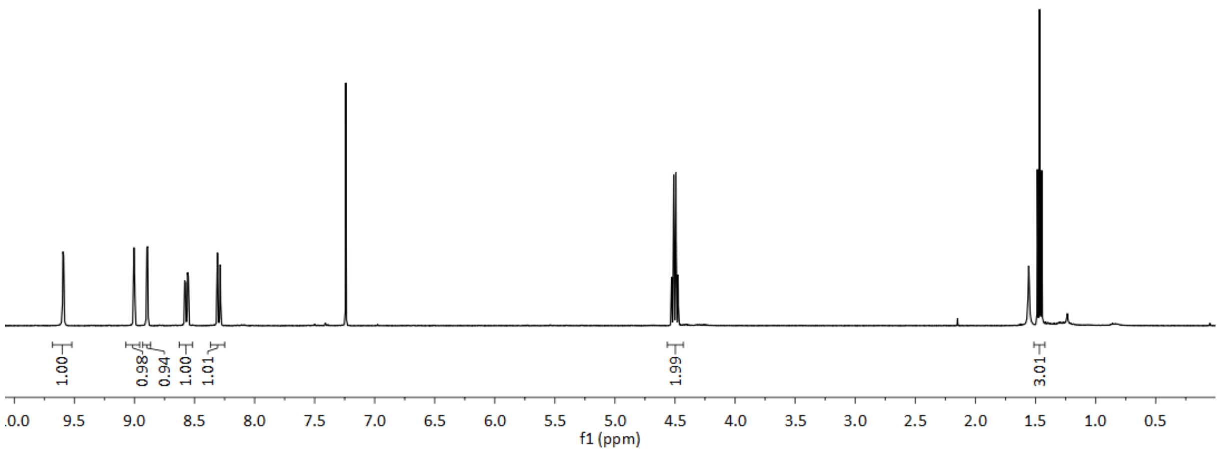

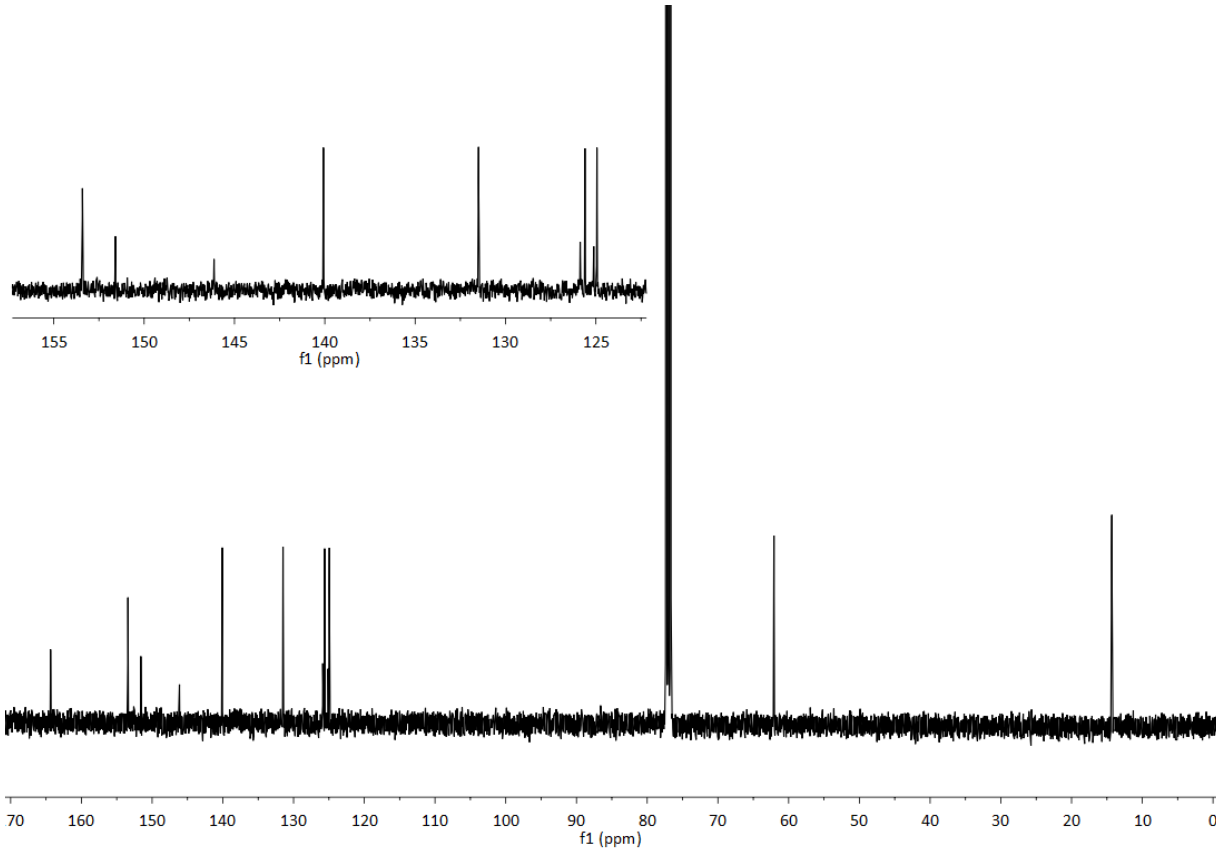

Ethyl 6-bromoquinoline-3-carboxylate. ^{1}H NMR ($CDCl_3$, 400 MHz) and ^{13}C NMR (100 MHz).

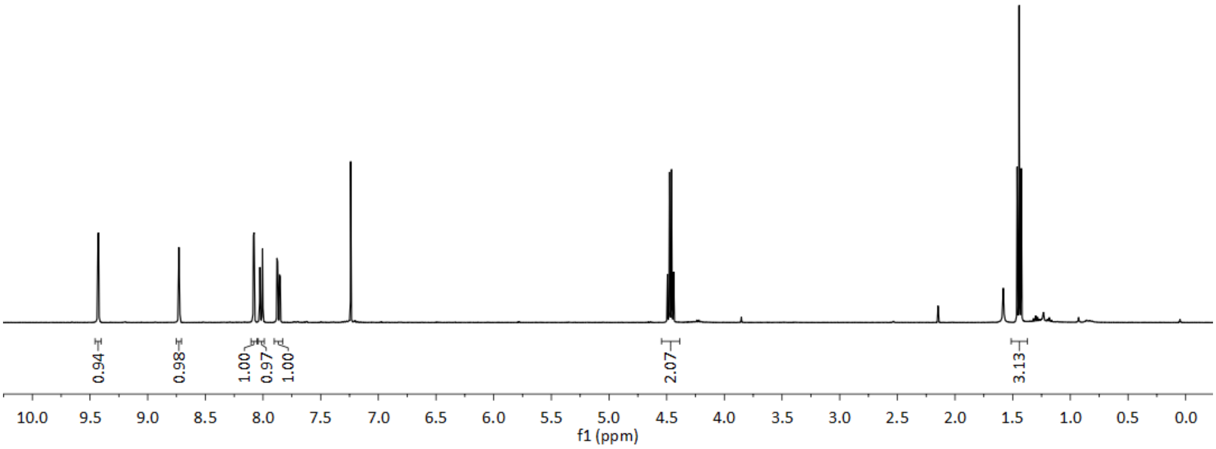

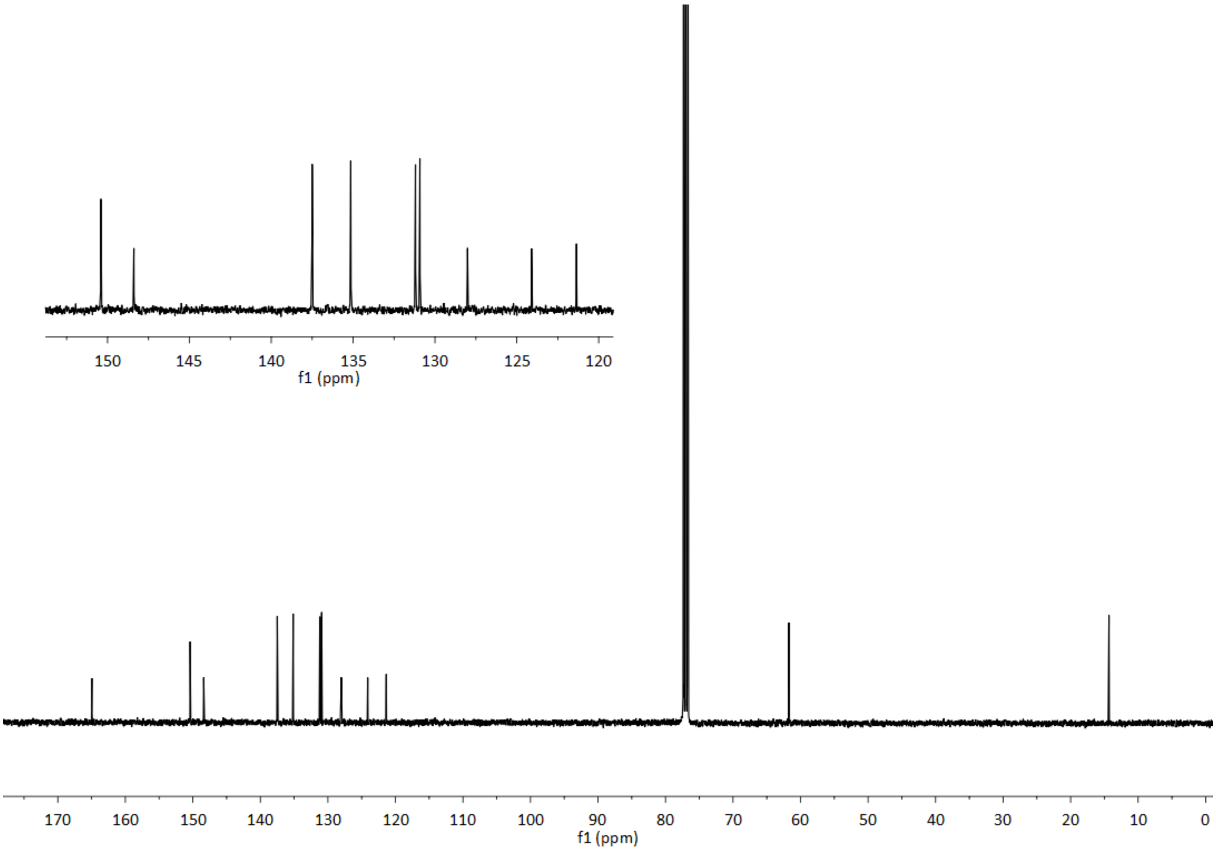

Ethyl 5-bromoquinoline-3-carboxylate. ^{1}H NMR ($CDCl_3$, 400 MHz) and ^{13}C NMR (100 MHz).

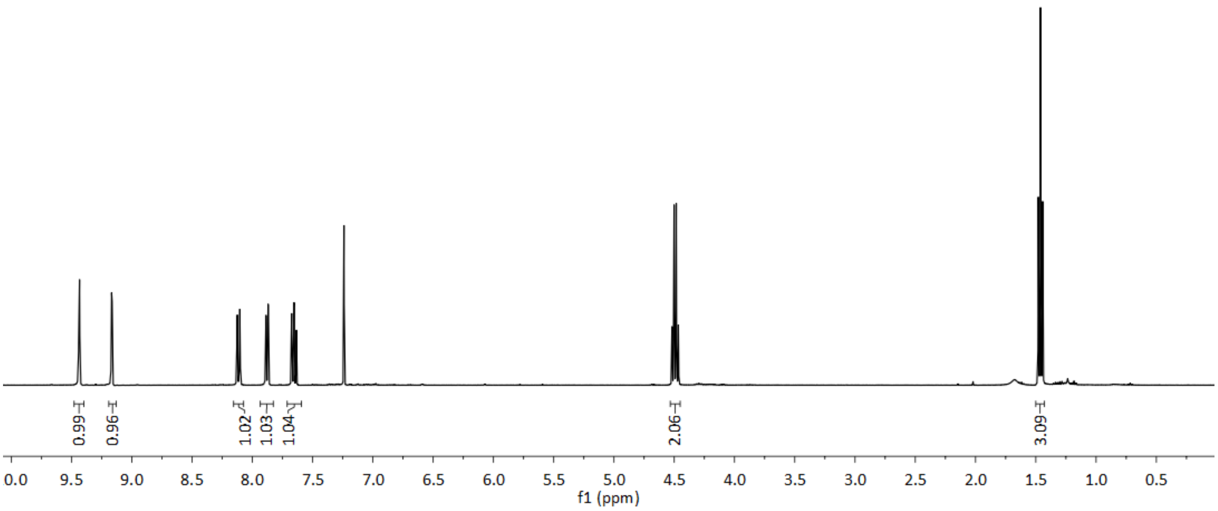

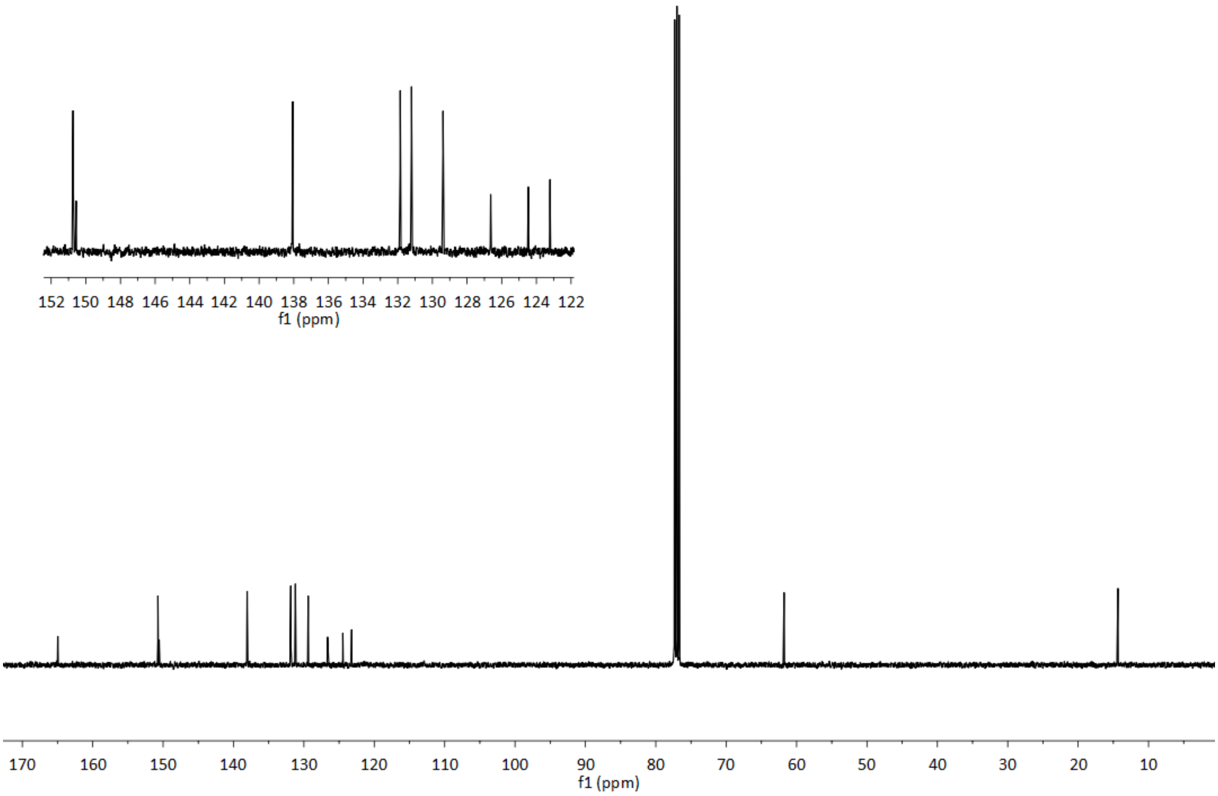

Ethyl 7-bromoquinoline-3-carboxylate. ^{1}H NMR ($CDCl_3$, 400 MHz) and ^{13}C NMR (100 MHz).

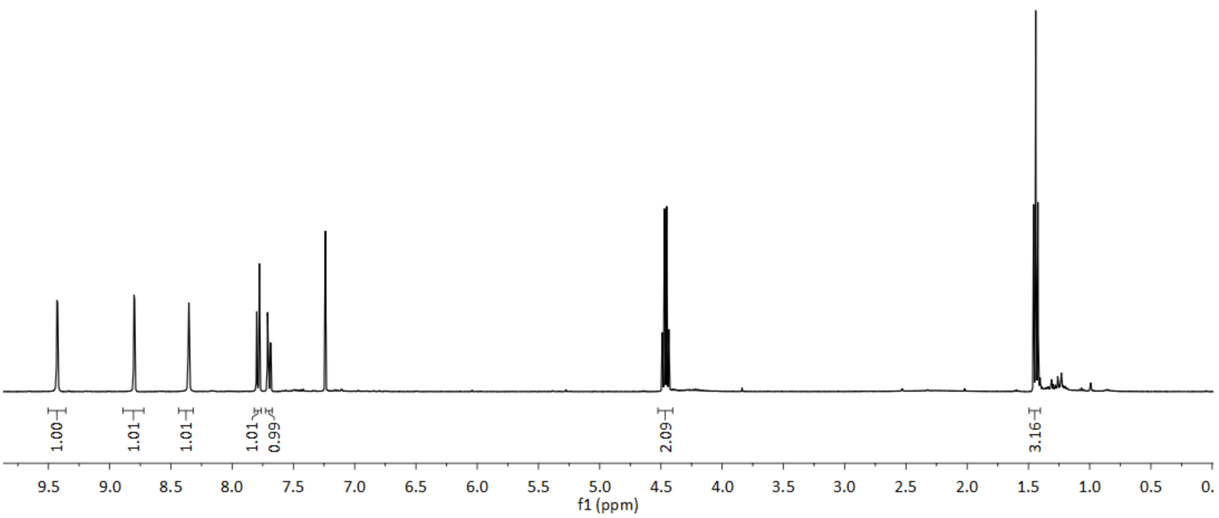

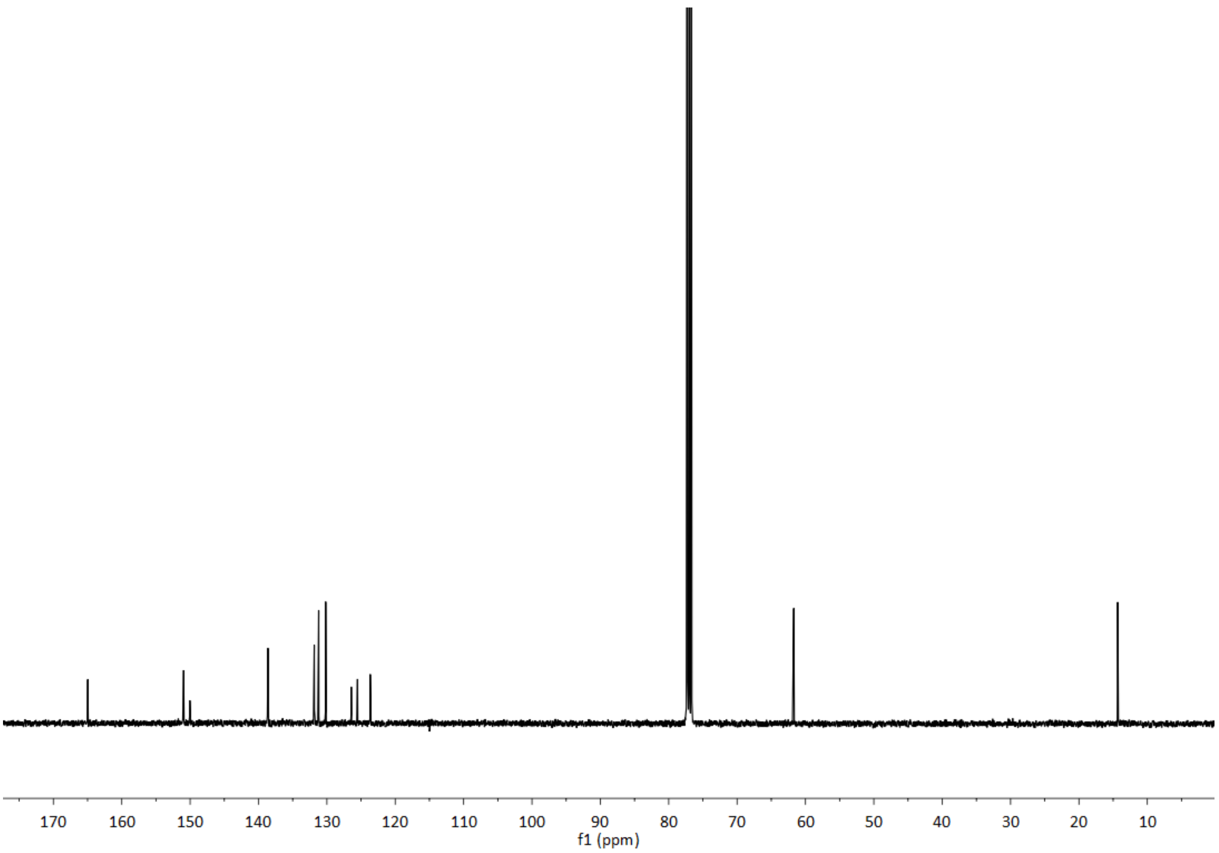

Ethyl 4-(2-methoxy-2-oxoethyl)quinoline-3-carboxylate. ^{1}H NMR ($CDCl_3$, 400 MHz) and ^{13}C NMR (100 MHz).

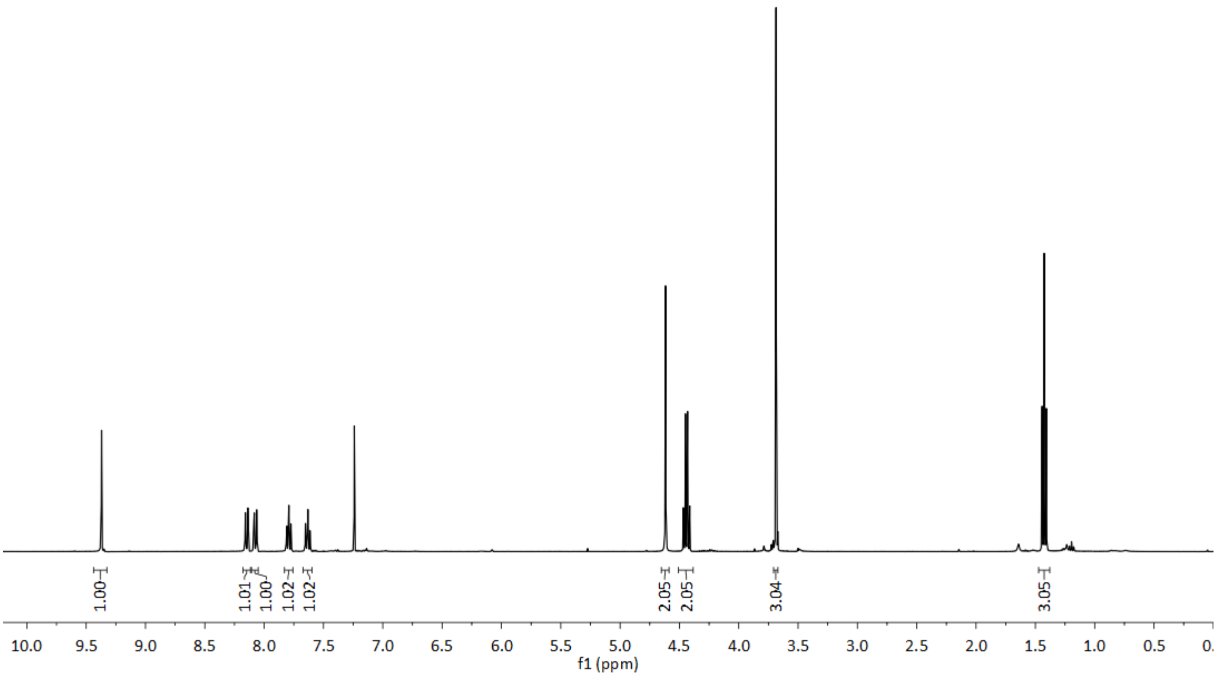

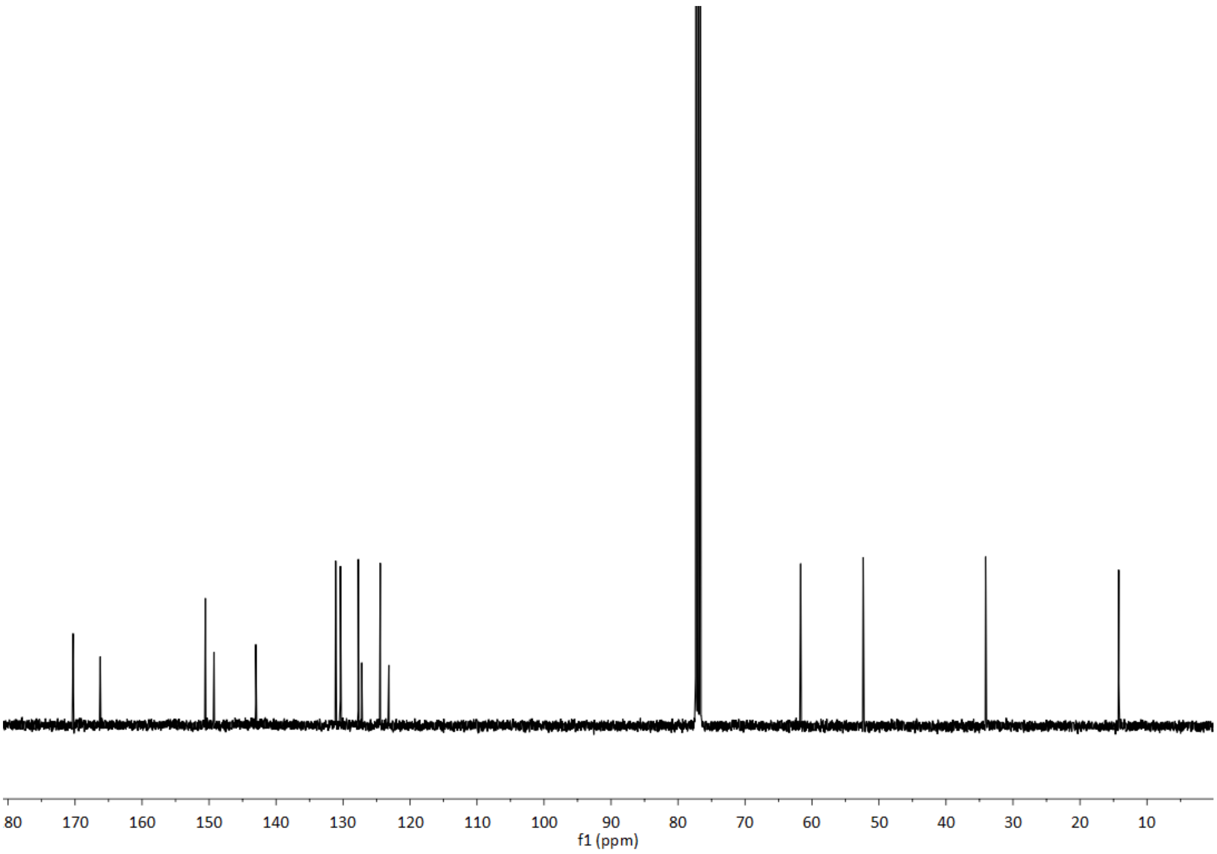

Ethyl 2,2-bis(1-methyl-1*H*-indol-3-yl)acetate. ^{1}H NMR ($CDCl_3$, 400 MHz) and ^{13}C NMR (100 MHz).

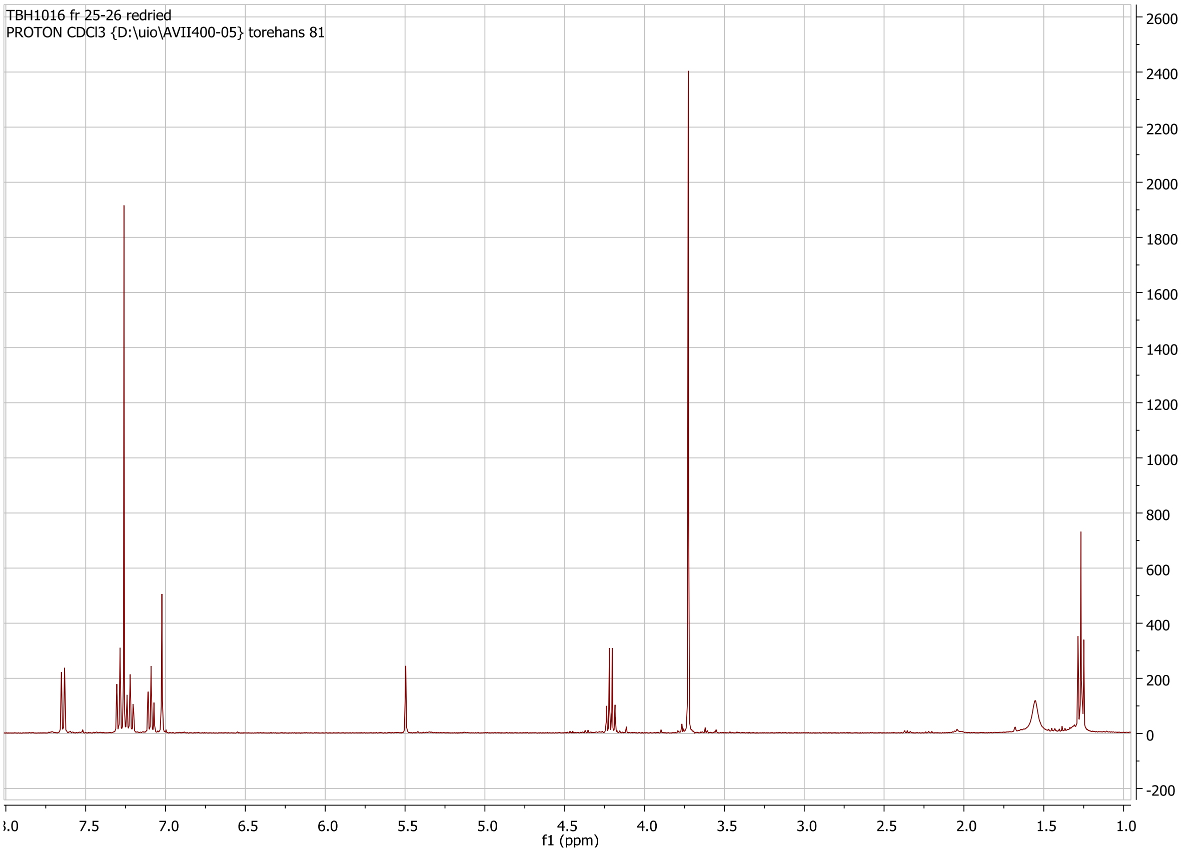

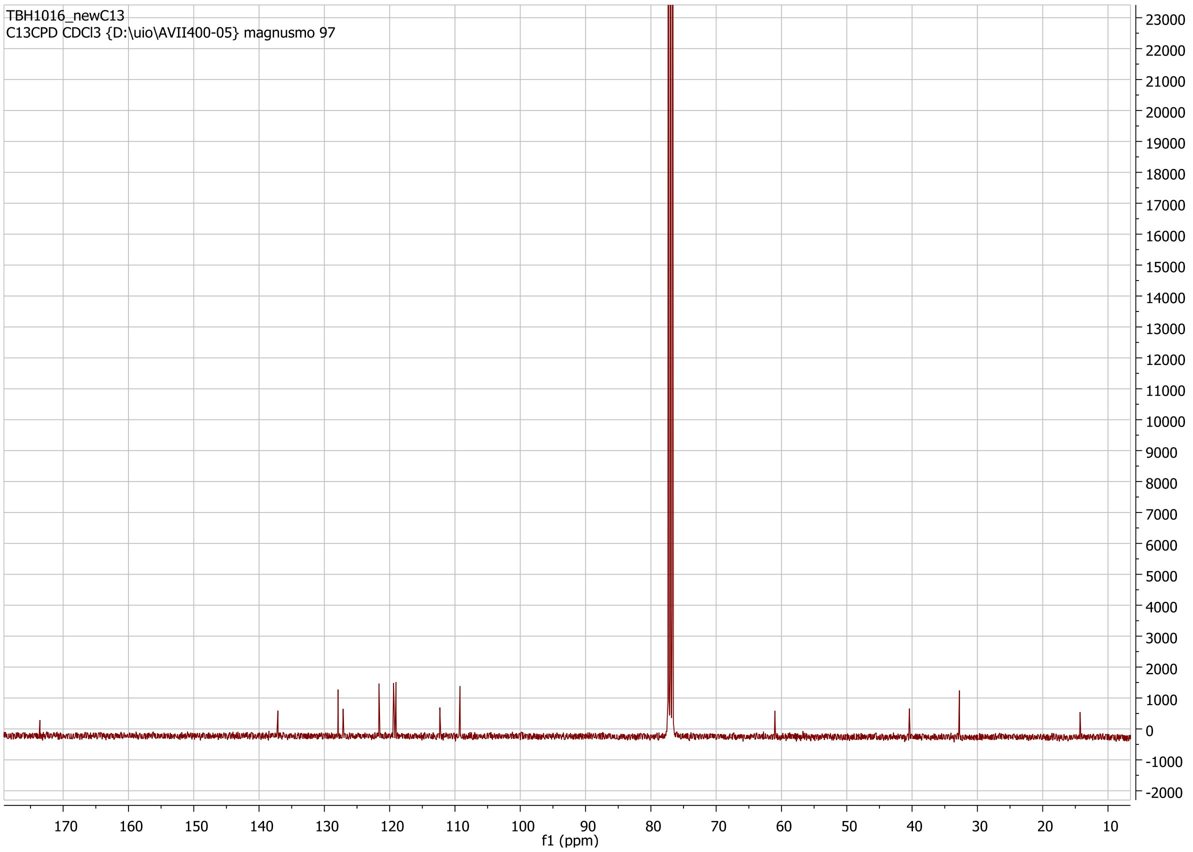

X-ray crystal structures

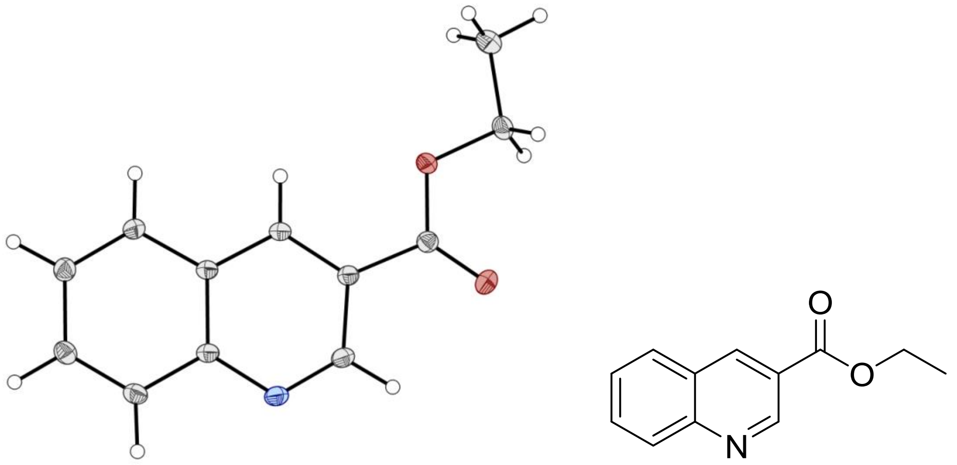

The ORTEP drawing from the X-ray crystal structure of ethyl quinoline-3-carboxylate.

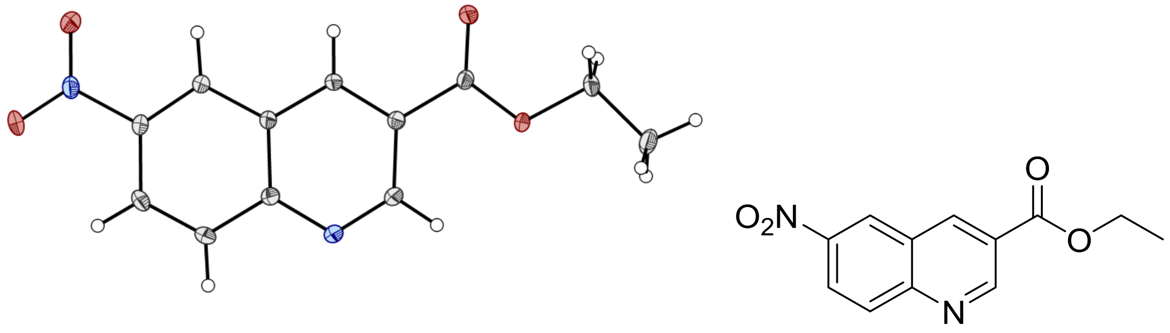

The ORTEP drawing from the X-ray crystal structure of ethyl 6-nitroquinoline-3-carboxylate.

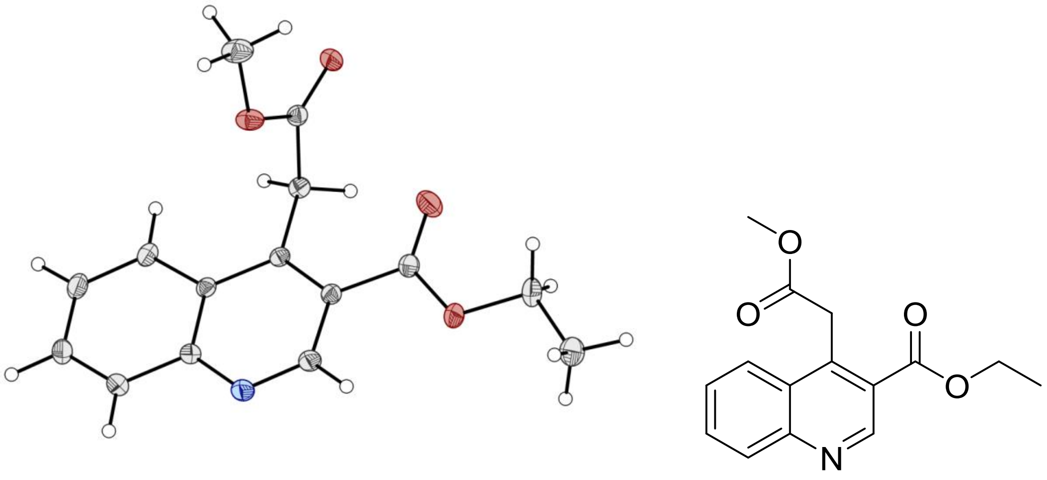

The ORTEP drawing from the X-ray crystal structure of ethyl 4-(2-methoxy-2-oxoethyl-quinoline-3-carboxylate.

References

1. H. Venkatesan, F. M. Hocutt, T. K. Jones, M. H. Rabinowitz, *J. Org. Chem.* **2010**, *75*, 3488-3491.

2. J. Tummatorn, C. Thongsornkleeb, S. Ruchirawat, T. Gettongsong, *Org. Biomol. Chem.* **2013**, *11*, 1463-1467.

3. H.-M. Dong, H.-H. Lu, L.-Q. Lu, C.-B. Chen, W.-J. Xiao, *Adv. Synth. Catal.* **2007**, *349*, 1597-1603.
